# Ferromagnetism in freestanding MoS_2_ nanosheets

**DOI:** 10.1186/1556-276X-8-129

**Published:** 2013-03-16

**Authors:** Daqiang Gao, Mingsu Si, Jinyun Li, Jing Zhang, Zhipeng Zhang, Zhaolong Yang, Desheng Xue

**Affiliations:** 1 Key Laboratory for Magnetism and Magnetic Materials of MOE, Lanzhou University, Lanzhou, 730000, People's Republic of China

**Keywords:** Ferromagnetism, MoS_2_ nanosheets, Exfoliation

## Abstract

Freestanding MoS_2_ nanosheets with different sizes were prepared through a simple exfoliated method by tuning the ultrasonic time in the organic solvent. Magnetic measurement results reveal the clear room-temperature ferromagnetism for all the MoS_2_ nanosheets, in contrast to the pristine MoS_2_ in its bulk form which shows diamagnetism only. Furthermore, results indicate that the saturation magnetizations of the nanosheets increase as the size decreases. Combining the X-ray photoelectron spectroscopy, transmission electron microscopy, and electron spin resonance results, it is suggested that the observed magnetization is related to the presence of edge spins on the edges of the nanosheets. These MoS_2_ nanosheets may find applications in nanodevices and spintronics by controlling the edge structures.

## Background

As a kind of layered semiconducting material, molybdenum disulfide (MoS_2_) has attracted much research interest due its unique physical, optical, and electrical properties correlated with its two-dimensional (2D) ultrathin atomic layer structure [1-4]. Unlike graphite and layered hexagonal BN (*h*-BN), the monolayer of MoS_2_ is composed of three atom layers: a Mo layer sandwiched between two S layers. The triple layers are stacked and held together through weak van der Waals interactions [5-10]. Recently, reports demonstrate strong photoluminescence emergence and anomalous lattice vibrations in single- and few-layered MoS_2_ films [5,6], which exemplify the evolution of the physical and structural properties in MoS_2_, due to the transition from a three-dimensional to a 2D configuration. Results also indicate that the single-layer MoS_2_ exhibits a high channel mobility (approximately 200 cm^2^ V^−1^ s^−1^) and current on/off ratio (1 × 10^8^) when it was used as the channel material in a field-effect transistor [7]. Most recently, it is proposed that the indirect band gap of bulk MoS_2_ with a magnitude of approximately 1.2 eV transforms gradually to a direct band gap of approximately 1.8 eV in single-layer samples [8,9], which is in contrast to pristine graphene with a band gap of about 0 eV and few-layered *h*-BN with a band gap of about 5.5 eV [10,11]. All these results imply that 2D MoS_2_ nanosheets have possible potential applications in electronics, optics, and semiconductor technologies as promising complements to graphene and *h*-BN [5-11].

Recently, based on first-principle calculations, lots of reports reveal the promising electronic properties of monolayer MoS_2_ nanosheets and nanoribbons, predicting their potential application in spintronic devices [12-15]. Calculation results indicate that MoS_2_-triple vacancy created in a single-layer MoS_2_ can give rise to a net magnetic moment, while other defects related with Mo and S atoms do not influence the nonmagnetic ground state [13]. Shidpour et al. performed the calculation on the sulfur vacancy-related magnetic properties on the S-edge with 50% and 100% coverage of MoS_2_ nanoribbons, showing that a vacancy on the S-edge with 50% coverage intensifies the magnetization of the edge of the MoS_2_ nanoribbon, but such a vacancy on the S-edge with 100% coverage causes this magnetic property to disappear [14]. Most recently, for the MoS_2_ nanoribbons, Pan et al. and Li et al. predicted that S-terminated zigzag nanoribbons are the most stable even without hydrogen saturation. MoS_2_ zigzag nanoribbons are metallic and ferromagnetic, and their conductivity may be semiconducting or half metallic by controlling the edge structures saturated with H atoms. The armchair nanoribbons are semiconducting and nonmagnetic, with band gaps increased by the hydrogen saturation of their edge states [15,16]. Inconsequently, Botello-Mendez et al. found that armchair nanoribbons could be metallic and exhibit a magnetic moment. Besides, when passivating with hydrogen, the armchair nanoribbons become semiconducting [17].

Though a lot of interesting magnetic properties of MoS_2_ nanosheets and nanoribbons had been predicted, the corresponding experimental realization on MoS_2_ nanosheets and nanoribbons has been at the nascent stage. The reason may be the difficulties in the synthesis methods because MoS_2_ tends to form zero-dimensional closed structures (fullerene-like nanoparticles) or one-dimensional nanotube structures during the experimental fabrications as well as lower crystalline structures [18-20]. What we know so far, the only experimental report on magnetism in MoS_2_ came from a study on MoS_2_ nanosheet film deposition on Si (100) and tantalum foil substrates synthesized using thermal evaporation method. A confirmatory test was also employed to rule out the samples' contaminants, where MoS_2_ nanotubes fabricated on an alumina template using the similar source and setup were tested to be nonmagnetic [21]. However, the interface between the film and substrate as well as the substrate itself could influence the local structures and, subsequently, the magnetic properties of the samples [22]. Therefore, synthesis and understanding of the edge-based magnetism in substrate-free MoS_2_ nanosheets or nanoribbons are very necessary, and a further sensitive experimental verification is required.

In this paper, solution exfoliation method was employed to fabricate the MoS_2_ nanosheets with different sizes [23]. The structure and the magnetic properties of these nanosheets were studied.

## Methods

MoS_2_ nanosheets were prepared through exfoliation of bulk MoS_2_ (purchased from the J&K Chemical, Beijing, China) with different times. In a typical synthesis progress, 0.5-g MoS_2_ powders were sonicated in *N*,*N*-dimethylformamide (DMF, 100 mL) to disperse the powder for 2, 4, 6, 8, and 10 h, respectively. After precipitation, the black dispersion was centrifuged at 2,000 rpm for about 20 min to remove the residual large-size MoS_2_ powders. Then, the remainder solution was centrifuged at 10,000 rpm for 1 h to obtain the black products. To remove the excess surfactant, the samples were repeatedly washed with ethanol and centrifuged. Finally, the samples were dried at 60°C in vacuum condition.

The morphologies of the samples were obtained by high-resolution transmission electron microscopy (HRTEM, Tecnai™ G2 F30, FEI, Hillsboro, OR, USA). X-ray diffraction (XRD, X'Pert PRO PHILIPS (PANalytical B.V., Almelo, The Netherlands) with CuK*α* radiation) and selected area electron diffraction (SAED) were employed to study the structure of the samples. The measurements of magnetic properties were made using the Quantum Design MPMS magnetometer (Quantum Design, Inc., San Diego, CA, USA) based on a superconducting quantum interference device (SQUID). The spectrometer at a microwave frequency of 8.984 GHz was used for electron spin resonance (ESR JEOL, JES-FA300, JEOL Ltd., Akishima, Tokyo, Japan) measurements. X-ray photoelectron spectroscopy (XPS, VG ESCALAB 210, Thermo VG Scientific, East Grinstead, UK) was utilized to determine the bonding characteristics and the composition of the samples. The vibration properties were characterized by Raman scattering spectra measurement, which was performed on a Jobin Yvon LabRam HR80 spectrometer (HORIBA Jobin Yvon Inc., Edison, NJ, USA; with a 325-nm line of Torus 50-mW diode-pumped solid-state laser (Laser Quantum, San Jose, CA, USA)) under backscattering geometry. The infrared absorption spectra of the samples were conducted with the KBr pellet method on a Fourier transform infrared spectrometer (FTIR; NEXUS 670, Thermo Nicolet Corp., Madison, WI, USA) in the range of 400 to 4,000 cm^−1^. Atomic force microscopy (AFM; Dimension 3100 with Nanoscope IIIa controller, Veeco, CA, USA) was used to confirm the layer number by measuring the thicknesses in tapping mode in air.

## Results and discussion

**Figure 1:**
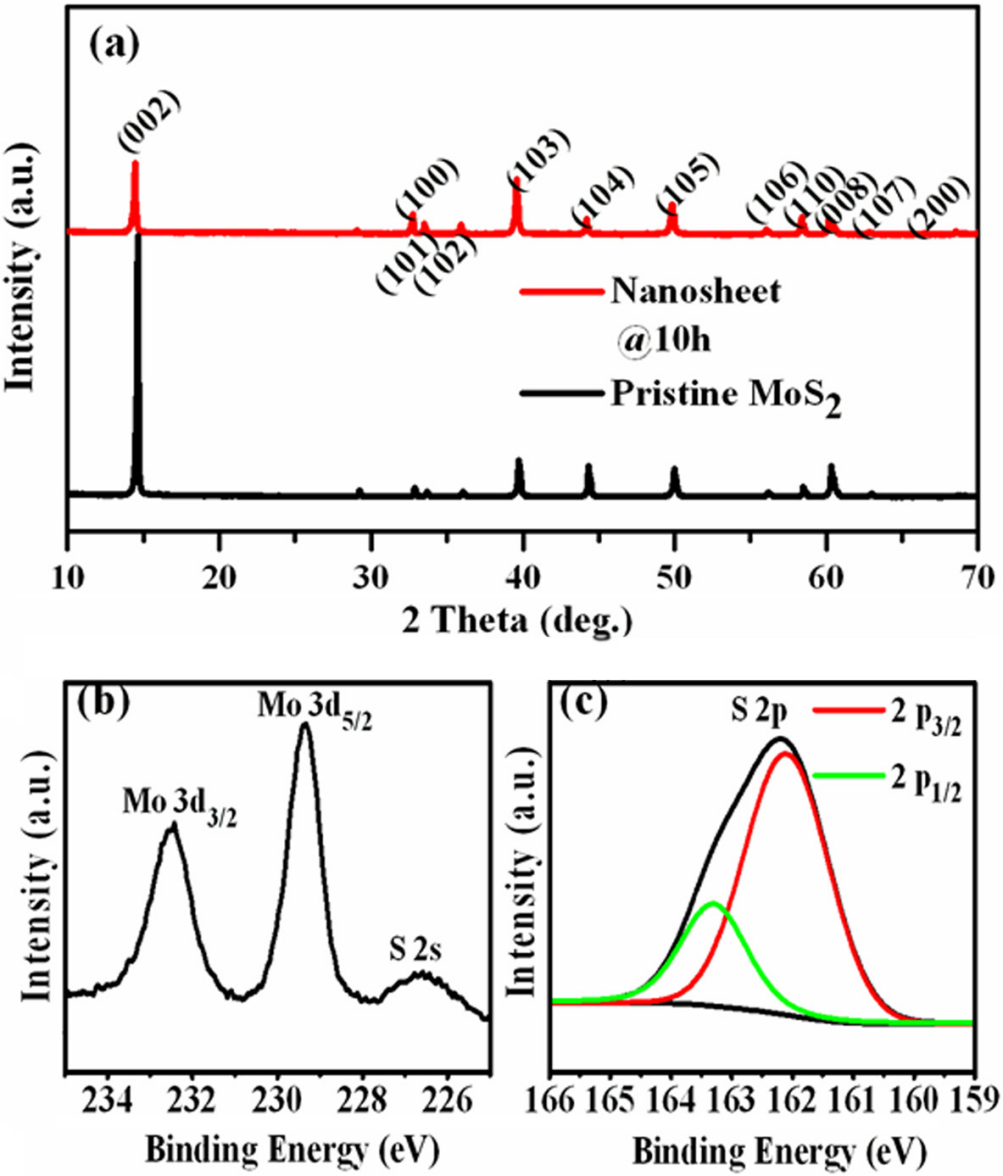
**XRD results and high-resolution XPS spectra. (a)** XRD results of MoS_2_ nanosheets and pristine MoS_2_ powders. High-resolution XPS spectra of **(b)** Mo 3*d* and **(c)** S 2*p* for the exfoliated MoS_2_ nanosheets (10 h)*.*

Sonication is known to peel off layered MoS_2_ from the pristine one due to interactions between solvent molecules and the surface of the pristine MoS_2_ powder [23]. The sonication time was tuned in our case to control the synthesis of the MoS_2_ nanosheets with different sizes and thicknesses. Typical XRD spectra of the pristine MoS_2_ used for exfoliation and the obtained sample are shown in Figure 1a; the reflection peaks can be assigned to the family lattice planes of hexagonal MoS_2_ (JCPDS card no.77-1716). After sonication in DMF for 10 h, the intensity of the (002) peak decreases abruptly, implying the formation of a few-layer MoS_2_ in the sample [24,25]. Furthermore, there is no other new phase introduced into the exfoliated MoS_2_ samples. The bonding characteristics and the composition of the exfoliated MoS_2_ samples were captured by XPS. Results indicate that the wide XPS spectra of the exfoliated MoS_2_ sample (10 h) show only signals arising from elements Mo and S besides element C (result is not shown here). The Mo 3*d* XPS spectrum of MoS_2_ nanosheets, reported in Figure 1b, shows two strong peaks at 229.3 and 232.5 eV, respectively, which are attributed to the doublet Mo 3*d*_5/2_ and Mo 3*d*_3/2_, while the peak at 226.6 eV can be indexed as S 2*s*. The peaks, corresponding to the S 2*p*_1/2_ and S 2*p*_3/2_ orbital of divalent sulfide ions (S^2−^), are observed at 163.3 and 162.1 eV (shown in Figure 1c). All these results are consistent with the reported values for the MoS_2_ crystal [26,27].

**Figure 2:**
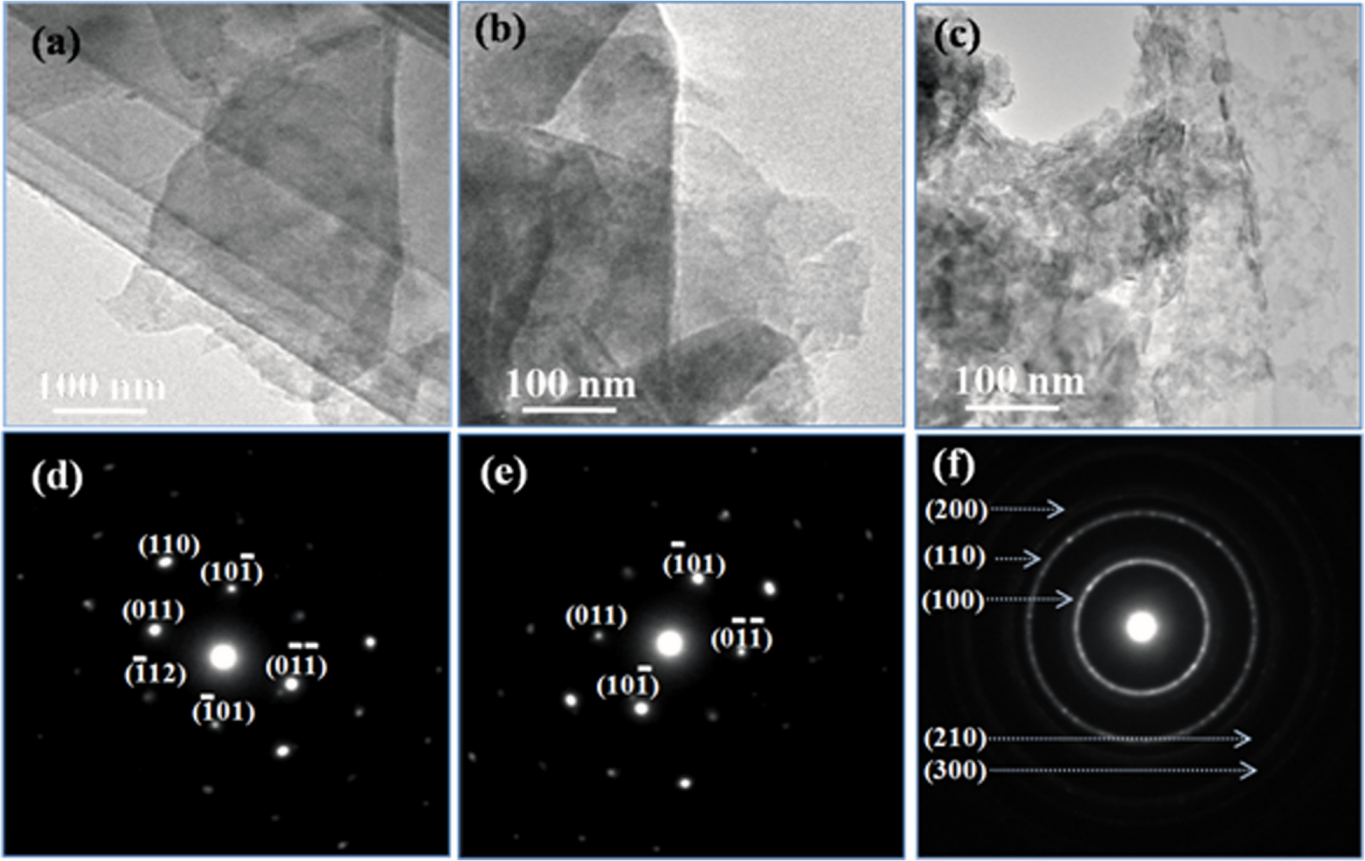
**TEM images of the exfoliated MoS**_**2 **_**nanosheets and their corresponding SAED results. (a**, **d)** 2 h, **(b**, **e)** 4 h, and **(c**, **f)** 10 h.

**Figure 3:**
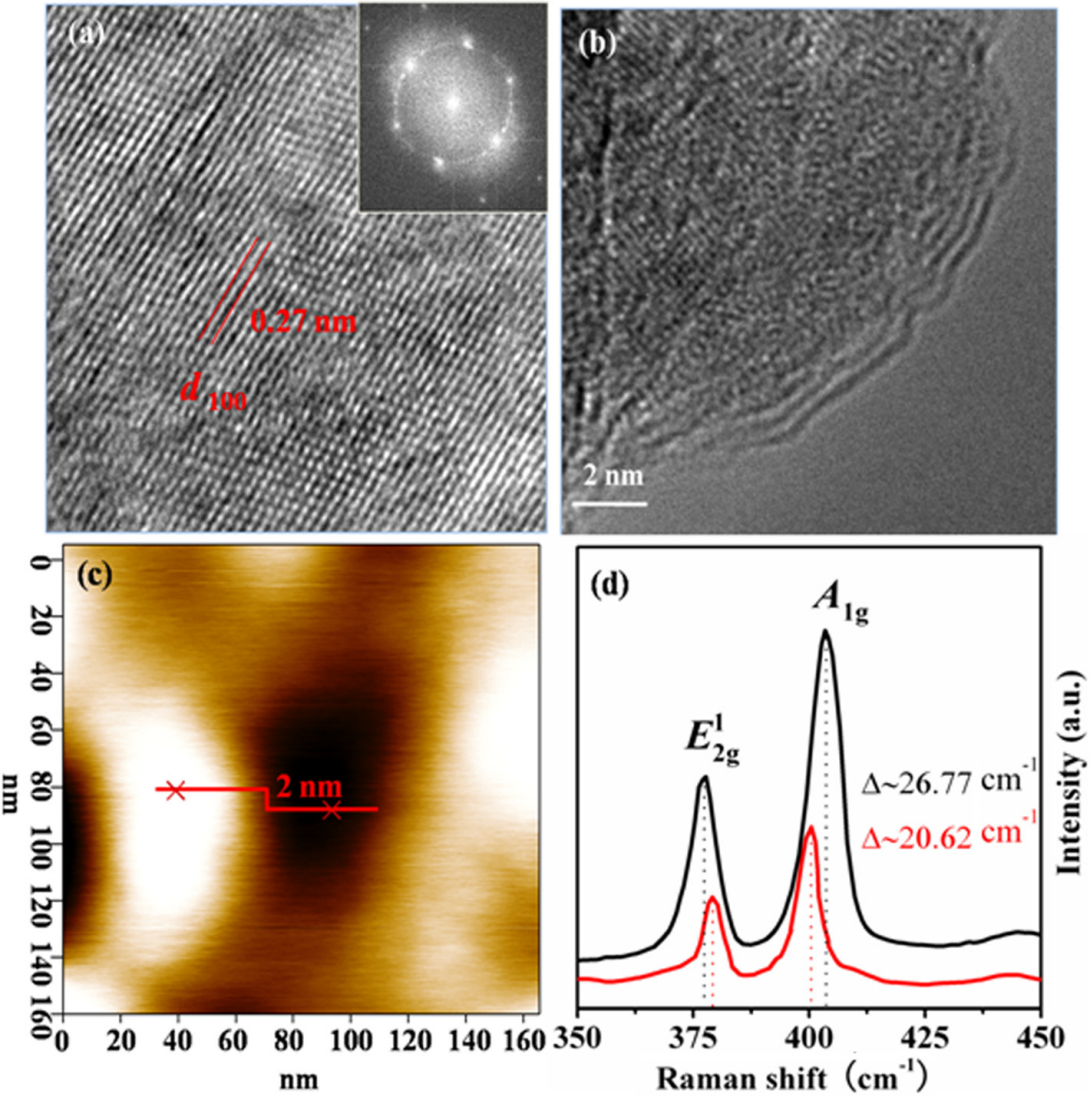
**HRTEM, TEM, and AFM images and Raman spectra of MoS**_**2 **_**nanosheets and MoS**_**2 **_**powder. (a)** The HRTEM image of exfoliated MoS_2_ nanosheets (10 h); the *d*_100_ is 0.27 nm. The inset is the FFT pattern of the sample. **(b)** Marginal TEM image of exfoliated MoS_2_ nanosheets (10 h). **(c)** Tapping mode AFM image of the exfoliated MoS_2_ nanosheets (10 h). **(d)** Raman spectra for the pristine MoS_2_ powder and exfoliated MoS_2_ nanosheets (10 h).

To better understand the exfoliation process and the nanosheet products, microscopic investigations were performed. TEM results for the exfoliated MoS_2_ sonicated at different times as shown in Figure 2a,b,c indicate that the samples have a sheet structure in irregular shapes, and the size of the nanosheets decreases gradually as the sonication time increases. Corresponding SAED results for the MoS_2_ nanosheets given in Figure 2d,e,f reveal the single crystal MoS_2_ in hexagonal structure. The HRTEM image in Figure 3a clearly reveals the periodic atom arrangement of the MoS_2_ nanosheets at a selected location, in which the interplanar spacing was measured to be 0.27 nm according to the periodic pattern in the lattice fringe image, matching up with that of the (100) facet of MoS_2_ (2.736 Å). HRTEM investigation in the edge areas was a common and direct method to determine the layer numbers microscopically [28]. In our case, as presented in Figure 3b, three to four dark and bright patterns can be readily identified for the exfoliated MoS_2_ nanosheet (10 h), indicating that the sample was stacked up with three to four single layers. For comparison, tapping mode AFM image of the MoS_2_ nanosheet (10 h) is shown in Figure 3c prominently; the thickness was measured to be only 2.0 nm, corresponding to the fundamental thickness of three single atomic layers of MoS_2_. Raman spectrum was used to confirm the few-layered MoS_2_ nanosheets. Generally, single-layer MoS_2_ exhibited strong bands at 384 and 400 cm^−1^, which are associated with the in-plane vibrational (*E*_2*g*_^1^) and the out-of-plane vibrational (*A*_1g_) modes, respectively [26]. As the layer number increased, a red shift of the (*E*_2*g*_^1^) band and a blueshift of the *A*_1g_ bands would be observed. Figure 3d shows the Raman spectra of the pristine MoS_2_ powder and the exfoliated MoS_2_ nansheets (sonicated in DMF for 10 h). Results indicate that the (*E*_2*g*_^1^) and *A*_1g_ bands for the pristine and MoS_2_ nanosheets are located at 376.90 and 379.21 cm^−1^, and 403.67 and 401.20 cm^−1^, respectively. The energy difference between two Raman peaks (Δ) can be used to identify the number of MoS_2_ layers. It can be seen that the Δ value obtained for the two samples is about 26.77 and about 20.62 cm^−1^, respectively, indicating the existence of the two to three layered MoS_2_ nanosheets after sonicating pristine MoS_2_ powders in DMF for about 10 h, which is the same as the TEM and AFM results.

**Figure 4:**
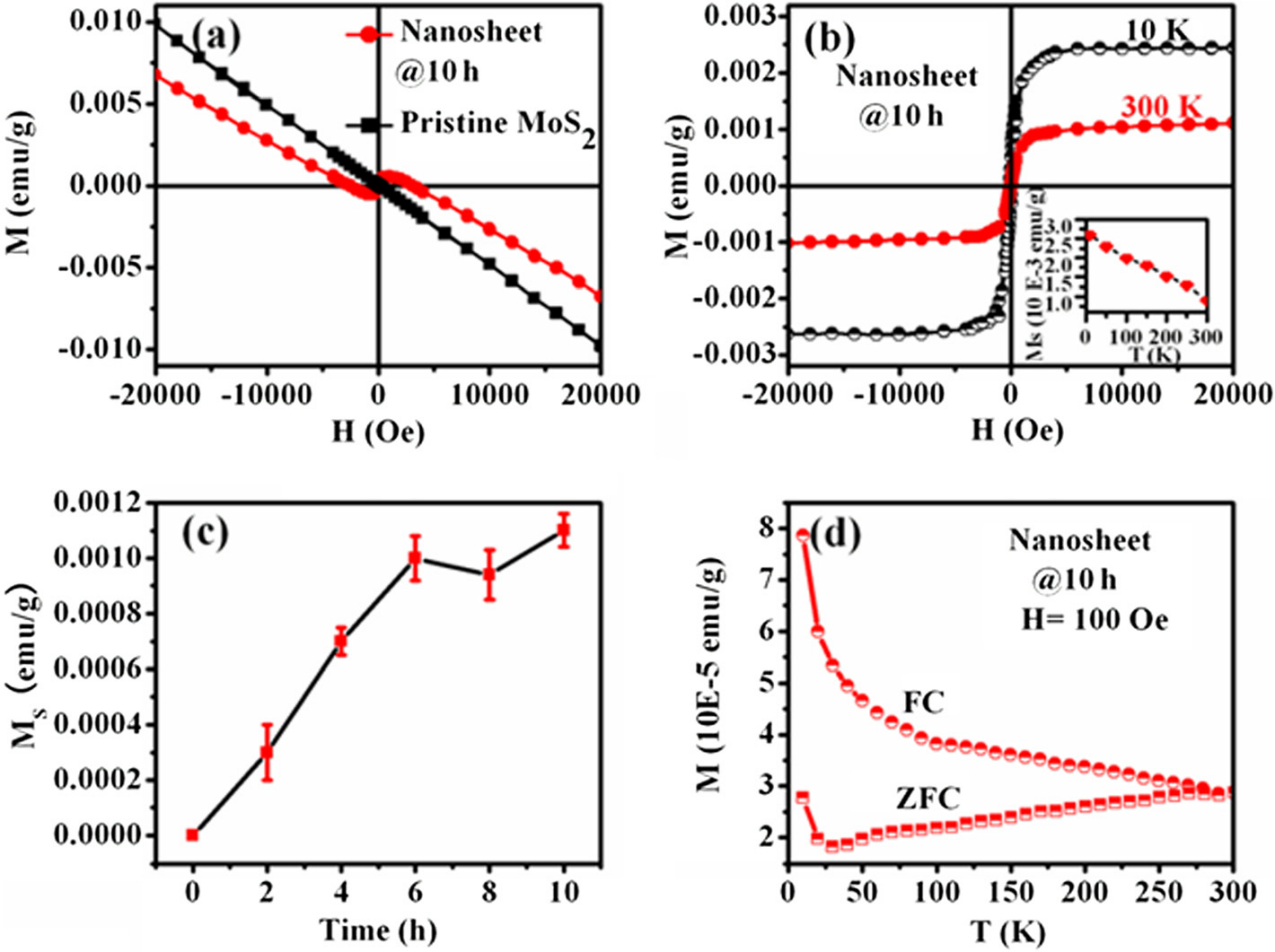
**Room-temperature *M*- *H *, ZFC, and FC curves. (a)** Room-temperature *M-H* curves for MoS_2_ pristine powders and nanosheets. **(b)***M-H* curves for MoS_2_ nanosheets measured at 10 and 300 K: the DM signals of the samples have been deducted. **(c)** The dependence of the saturation magnetization of the MoS_2_ nanosheets on sonication time. **(d)** The ZFC and FC curves for the exfoliated MoS_2_ nanosheets sonicated in DMF for 10 h.

**Figure 5:**
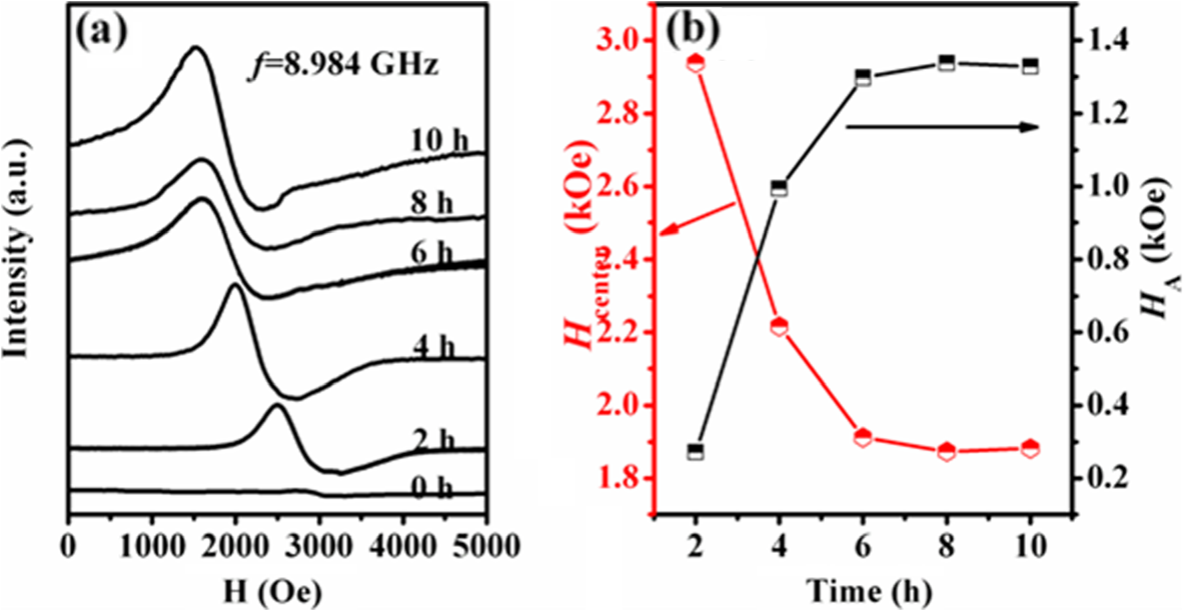
**ESR spectra and dependence of *H***_**center **_**and *H***_**A **_**on the sonication time. (a)** Room-temperature ESR spectra for MoS_2_ pristine powders and nanosheets. **(b)** Dependence of resonance center field and the anisotropy field of MoS_2_ nanosheets on the sonication time.

TEM results indicate that few-layered MoS_2_ nanosheets can be obtained after sonicating pristine MoS_2_ powders in DMF with different times; at the same time, the size (the lateral dimension for the nanosheets) of the nanosheets decreases gradually, which motivated us to carry out a comparative study on the size-property correlation magnetic properties of the MoS_2_ nanosheets. Figure 4a shows the magnetization versus magnetic field (*M*-*H*) curves for the pristine MoS_2_ powders and the exfoliated MoS_2_ nanosheets (sonicated in DMF for 10 h). As can be seen, besides the diamagnetic (DM) signal in the high-field region, the exfoliated MoS_2_ nanosheets show the ferromagnetism (FM) signal in lower field region as well, compared to the pristine MoS_2_ powders which shows the DM signal only. After deducting the DM signal, the measured saturation magnetizations (*M*_s_) for the MoS_2_ nanosheets (10 h) are 0.0025 and 0.0011 emu/g at 10 and 300 K, respectively (Figure 4b), which are comparable to other dopant-free diluted magnetic semiconductors [29,30]. Dependence of the *M*_s_ on ultrasonic time of the obtained MoS_2_ nanosheets is shown in Figure 4c. Results indicate that the *M*_s_ of the obtained MoS_2_ nanosheets increases gradually as the ultrasonic time increases, and then become invariable when the ultrasonic time exceeds 6 h. Combining with the TEM results, it can be concluded that the FM increases as the size for the nanosheets decreases. Zero-field-cooled (ZFC) and field-cooled (FC) measurements are performed on the sample which has the maximum *M*_s_, and the results are shown in Figure 4d. Results indicate that the FC curve exhibits an obvious deviation from the ZFC curve until 300 K, revealing that the Curie temperature of the sample is 300 K at least. Other exfoliated MoS_2_ nanosheets show the same ZFC and FC results, and the data are not shown here. Room-temperature ESR results shown in Figure 5a give further evidence for the FM of the exfoliated MoS_2_ nanosheets. Besides the pristine MoS_2_ powder, all the exfoliated MoS_2_ nanosheets have obvious ferromagnetic resonance signals. At the same time, the resonance center field (*H*_center_) for the MoS_2_ nanosheets shifts to a lower value as the size of the nanosheets decreases, revealing the enhanced FM. It can be understood from the condition for resonance in the presence of anisotropy field (*H*_A_): *hf*/*μ*_B_*g* = *H*_center_ + *H*_*A*_, where *h* is the Plank's constant, *g* ≈ 2 for a free electron, *f* (8.984 GHz) is the fixed frequency of the applied microwave magnetic field, and μ_*B*_ is the Bohr magnetron, respectively [31]. The data in Figure 5b suggest an increase in anisotropy *H*_A_ with a decreasing size of the nanosheets, which corresponds to the magnetic results of SQUID.

**Figure 6:**
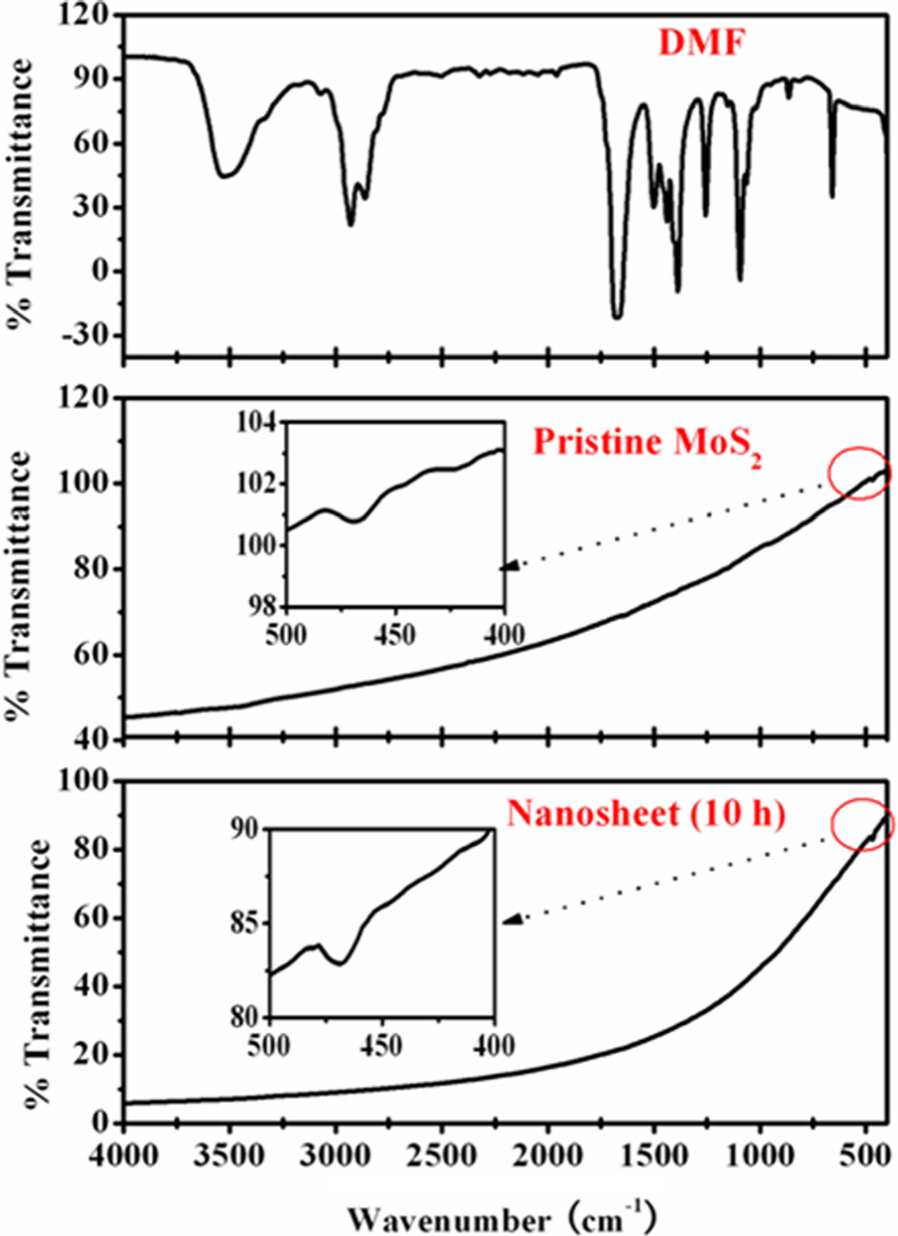
**FTIR patterns.** FTIR patterns of the solution DMF, the pristine MoS_2_ powder, and the MoS_2_ nanosheets sonicated in DMF for 10 h.

Recent calculation results indicate that the absorption of a nonmetal element on the surface of low-dimensional systems can induce a local magnetic moment [32]. Because our samples of MoS_2_ nanosheets are obtained by sonicating in the solution of DMF for a long time, whether the experiment progress can lead to the absorption of nonmetal elements in the samples needs to be verified. Here, FTIR measurement was applied in the range of 400 to 4,000 cm^−1^ to study the chemical compositions and bonds of the samples (shown in Figure 6). Results indicate that there is only one weak absorption peak at 474.1 cm^−1^ for the pristine MoS_2_ powder, which can be ascribed to characteristic Mo-S stretching vibration mode of MoS_2_. Note that the exfoliated MoS_2_ nanosheets has the same FTIR result as the pristine MoS_2_ powder, indicating that there is no absorbed element induced during the experiment progress, and the observed FM in our case is not caused by the surface absorption. Furthermore, contamination of magnetic elements is a possible source of the observed FM in nonmagnetic materials, so it is important to rule out such possibility. In our case, first, XRD, HRTEM, and XPS results show no other phases and the possible impurities in the samples; second, the sensitivity of *M*_s_ values to the ultrasonic time seen above (Figure 4c), changing by almost ten orders of magnitude, may not be attributed to the possible contamination in the samples, especially when the MoS_2_ nanosheets were obtained by keeping all other parameters identical besides the sonication time. In addition, the ZFC curve for the sample having the maximum *M*_s_ shows no blocking temperature in the range of 5 to 300 K, indicating that there is no ferromagnetic contamination in the sample. Therefore, it is suggested that the observable FM in MoS_2_ nanosheets is not due to contaminants.

First-principle calculation results reveal the nonmagnetic properties for the infinitely single-layered MoS_2_, and the FM in MoS_2_ nanoribbons is considered to be dominated by its zigzag edges [15,16], In addition, the unit magnetic moment of MoS_2_ nanoribbons (magnetic moment per MoS_2_ molecular formula) decreases gradually with increasing ribbon width, implying that the magnetism of MoS_2_ nanoribbons gets weaker and weaker as the ribbon width increases and disappears finally in the infinitely single-layered MoS_2_ and bulk. In our case, the size of the nanosheets decreases gradually with increasing ultrasonic time in the organic solvent DMF, and the enhancement of the FM for the nanosheets was also observed as the size decreases. This is because the magnetic behavior in MoS_2_ nanosheets results from the unsaturated edge atoms, and the ratio of edge atoms vs. total atoms increases dramatically as the size decreases. Therefore, the observed FM in MoS_2_ nanosheets is considered to be related to the intrinsic morphology of the materials.

## Conclusion

In summary, MoS_2_ nanosheets of different sizes were fabricated by exfoliation of bulk MoS_2_ in DMF solution. Magnetic measurements indicate that all the fabricated MoS_2_ nanosheets show obvious RT FM, and the enhanced FM was observed as the size of the nanosheets decreases. The intrinsic room-temperature FM for the samples is considered to be related to the presence of edge spins on the edges of the nanosheets.

## Competing interests

The authors declare that they have no competing interests.

## Authors' contributions

DG participated in all of the measurements and data analysis and drafted the manuscript. DX conceived and designed the manuscript. ZY and ZZ prepared all the samples and carried out the XPS measurements and data analysis. JZ participated in the SQUID measurements. MS and JL carried out the calculation part and data analysis. All authors were involved in the revision of the manuscript and read and approved the final manuscript.
